# Effect of sodium stearoyl-2-lactylate supplementation on lactation performance, blood-biochemical profile, and economic efficacy of mid-lactation Holstein cows

**DOI:** 10.5713/ajas.18.0367

**Published:** 2018-07-26

**Authors:** Eun Tae Kim, Sang Suk Lee, Ji Hoon Lee, Jin Suk Jeong, Shin Ja Lee, Joon Jeong, Jong Kook Park, Beom Young Park, Sang Bum Kim, Ha Yeon Jeong, Kwang Seok Ki, Chang Weon Choi, Chang Hyun Kim, Jin Wook Kim, Sung Sill Lee

**Affiliations:** 1 National Institute of Animal Science, Rural Development Administration, Cheonan 31000, Korea; 2 Department of Animal Science and Technology, Sunchon National University, Suncheon 57922, Korea; 3 Division of Applied Life Science (BK21 Plus), Gyeongsang National University, IALS, Jinju 52825, Korea; 4 Livestock Research Institute, National Agricultural Cooperative Federation, Anseong 17558, Korea; 5 Department of Animal Resources, Daegu University, Gyeongsan 38453, Korea; 6 Department of Animal Life and Environment Science, Hankyong National University, Anseong 17579, Korea

**Keywords:** Sodium Stearoyl Lactylate, Holstein Cows, Blood Parameters, Economic Efficacy, Lactation Performance

## Abstract

**Objective:**

This study was done to evaluate the effect of sodium stearoyl-2-lactylate (SSL) supplementation in a total mixed ration (TMR) on the lactation performance, blood parameters, and economic efficacy of mid-lactation Holstein cows.

**Methods:**

Twenty-four cows (body weight 647±11.7 kg) were randomly divided into 4 treatment groups, with six cows per group. The dietary treatments were as follows: basal diet (CON); CON+17.5 g of top dressed SSL (treatment [TRT] 0.05); CON+35 g of SSL (TRT 0.1); and CON+70 g of SSL (TRT 0.2) per 35 kg TMR.

**Results:**

The highest level of SSL supplementation (TRT 0.2) significantly improved milk yield during the second period compared to the TRT 0.05 group (5 to 8 wks; 33.28 vs 31.09 kg/d), during the third period compared to both the CON and TRT 0.05 groups (p<0.05) (9 to 13 wks; 32.59 vs 30.64 and 30.01 kg/d) and during the overall experimental period compared to both the CON and TRT 0.05 groups (p<0.05) (1 to 13 wks; 33.43 vs 32.06 and 31.40 kg/d), respectively. No negative effects on hematological or biochemical parameters were observed due to SSL supplementation. Considering both the milk fat and protein content, the total milk price was set at 1,073.60 (TRT 0.05), 1,085.60 (TRT 0.1), 1,086.10 (TRT 0.2), and 1,064.20 (CON) won/L, with consequent total milk profits of −1.7%, 5.4%, and 3.5% for the TRT 0.05, TRT 0.1, and TRT 0.2 diet, respectively, compared to those in the CON diet.

**Conclusion:**

The milk sales revenue related to SSL supplementation of the TRT 0.1 diet was increased by up to 5.4% compared to the milk sales revenue of the CON diet. Therefore, 0.1% SSL supplementation might be effective and profitable during the mid-lactation period of cows, without producing adverse effects.

## INTRODUCTION

Recent generations of cows have been improved to produce high lactation yields through selective breeding and enhanced animal management. However, nutritional and physical imbalances during the early lactation period can be easily occurred in dairy cows. Feeding management for high-producing dairy cows during pre-calving and post-calving periods impacts animal performance [1]. Enhancing lipid use can improve feed efficacy and animal productivity while reducing feed costs, as fat is an essential nutrient with the highest energy density among the major macronutrients [2]. Lipids are commonly used to improve feed energy for high yield cows, and adding them to the feed has many benefits, such as improved fat-soluble nutrient absorption (i.e., essential fatty acids, vitamin A, D, E, and K), increased palatability, and reduction of feed dust [3]. Many studies have investigated methods for improving lipid use in ruminants [4–6]. Some studies have focused on surfactant supplementation for emulsification [7–10]; for example, beneficial effects of surfactants have been observed on ruminal fermentation as well as animal performance. However, the response to surfactants is not always positive, as shown by Hristov et al [11] and Wang et al [12]. Dietary lipids must be broken down into smaller structures to be absorbed in the small intestine. Zinn [13] and Cho et al [14] suggested that increased surface area and micelle number can help determine the rate and extent of lipid hydrolysis, which can improve lipid digestibility and fat yield in cattle supplemented with surfactants. Furthermore, lipids in an oil-in-water state within the small intestine undergo a different absorption process than hydrophilic carbohydrates and proteins. Thus, to efficiently digest and absorb lipids, oil-in-water system surfactants, such as sodium stearoyl lactylate (SSL), act as positive emulsifiers compared to water-in-oil system surfactants, such as lecithin [15], and can resolve immiscibility and provide stability to an oil-water system. The hydrophilic-lipophilic balance in SSL is between 10 and 12, which indicates that SSL is an excellent emulsifier to facilitate the formation of fat in water emulsions for lipid digestion in the small intestine, thus leading to emulsion stability [16]. Therefore, this study was under taken to ascertain the effect of SSL supplementation in a total mixed ration (TMR) diet on the lactation performance, blood parameters, and economic efficacy of mid-lactation Holstein cows.

## MATERIALS AND METHODS

### Animal care

All experimental procedures performed in this study were approved by the Animal Care Committee of Gyeongsang National University (Jinju, Gyeongsangnam-do, Republic of Korea).

### Animals, diets and management

A total of twenty-four lactating Holsteins cows, with an initial body weight of 647±11.7 kg (mean±standard deviation) and parity of 2.3±0.30 were randomly divided into 4 treatment groups with six cows per treatment. The dietary treatments included the following: CON, basal diet; treatment (TRT) 0.05, CON+17.5 g of top dressed SSL; TRT 0.1, CON+35 g of SSL; and TRT 0.2, CON+70 g of SSL per 35 kg of TMR. All experimental diets were formulated to meet or exceed NRC [17] recommendations for a 650-kg cow producing 33 kg/d of milk, with 4.0% fat and 3.4% protein, regardless of treatment. Cows were fed twice per day at 06:00 h and 18:00 h in their stalls and supplemented with SSL as the top dressing of the basal ration. After a 7-day adaptation period, the animals were exposed to the different treatments for the entire experimental period. The animals were placed in an environmentally regulated facility and provided *ad libitum* access to water throughout the 98-day experimental period. Experimental diet samples were dried in a forced-air oven at 130°C for 2 h, after being finely ground through a 2-mm screen in a Wiley mill (Model 4, Thomas Scientific, Swedesboro, NJ, USA). The ground samples were analyzed to determine the amounts of dry matter, crude protein, Ca, and P according to the AOAC procedure [18]. Ether extraction was performed by the diethyl ether extraction method using a Buchi B-811 Universal Extraction System (Buchi, Flawil, Switzerland). Crude fiber was analyzed by the filter bag technique using the ANKOM 220 Fiber Analyzer (Mill Tech, Seongnam, Korea), and ash was analyzed by an electric muffle furnace using a KMF-500 (Lab Corporation, Seoul, Korea) after burning at 550°C. The concentration of neutral detergent fiber, which includes cellulose, hemicellulose and lignin as the major components, corrected for residual ash, was determined with thermostable alpha-amylase using the method of Van Soest et al [19], while the concentration of acid detergent fiber (Method 973.18), with inclusion of residual ash, was determined according to the AOAC procedure [18]. The net energy of lactation is the energy required for lactation and maintenance [17], and this was calculated using the metabolic energy from the apparent total tract digestibility of the digestible protein, fat and carbohydrates. The characteristics of the experimental animals, TMR ingredients and chemical compositions are shown in Table 1.

### Sampling, measurements and chemical analysis

The animals were milked twice per day at 05:00 and 17:00 for the first 13 weeks, and the daily milk yield was recorded daily using a Milk Meter (TRU-TEST Ltd., Central Otago, New Zealand). Milk samples from both morning and evening milking were collected once every 3 weeks and analyzed to determine fat, protein, lactose, solids-non-fat (SNF), and milk urea nitrogen (MUN) content using a Speedy Lab (Astorilab, Poncarale, Italy), as shown in Table 2. Blood samples were obtained by direct venipuncture of the jugular vein at the end of the experimental period, prior to the morning feeding. Whole blood samples (6 mL) were collected in 10 mL BD vacuum tubes with sodium heparin (Becton and Dickinson, NJ, USA). After clotting at 4°C for 24 h, serum analysis samples were centrifuged at 2,500×g at 4°C for 30 min, separated, and stored at −70°C until further analysis. Blood samples were analyzed using a Hitachi 7020 automatic blood analyzer (Hitachi, Tokyo, Japan). Whole blood samples were used to measure hematological parameters including the white blood cell (WBC) count, red blood cell (RBC) count, hemoglobin content and hematocrit value. Plasma blood samples were used to measure the chemical composition of the plasma including the total protein, albumin, glucose, aspartate aminotransferase, alanine aminotransferase (ALT), gamma glutamyl transferase, blood urea nitrogen, creatinine, total triglycerides, non-esterified fatty acid, and cholesterol concentrations [20,21].

### Economic efficacy analysis

The TMR price as fed dry matter was set at 600 won/kg, and lactating feed was set to 764 won/kg with 87% total digestible nutrients and 18% crude protein based on the standard of milk pricing concept from the Korea Dairy Committee [22]. The economic analysis was conducted based on the cost of feed intake and the price of milk yield. The total feed and milk costs did not include basic costs such as labor, equipment, materials, energy and water.

### Statistical analysis

All data for cows within each treatment group were averaged and analyzed using the PROC GLM procedure in SAS [23] with the following statistical model: Y_ij_ = μ+TRT_i_+e_ij_, where Y_ij_ is an observation on the dependent variable ij, μ is the overall population mean, TRT_i_ is the fixed effect of treatments, and e_ij_ is the random error associated with the observation ij. Duncan’s multiple range test [24] was used to determine significant differences among the mean values of the treatments. Variability in the data was expressed as the standard error of the mean; a value of p<0.05 was considered statistically significant, and a value of p<0.10 was considered a tendency.

## RESULTS AND DISCUSSION

### Lactation performance

The milk yield and milk composition of mid-lactation cows supplemented with different amounts of SSL are shown in Table 2. The highest level of SSL supplementation (TRT 0.2) significantly improved milk yield during the second period compared to the TRT 0.05 group (5 to 8 wks; 33.28 vs 31.09 kg/d), during the third period compared to both the CON and TRT 0.05 groups (p<0.05) (9 to 13 wks; 32.59 vs 30.64 and 30.01 kg/d) and during the overall experimental period compared to both the CON and TRT 0.05 groups (p<0.05) (1 to 13 wks; 33.43 vs 32.06 and 31.40 kg/d), respectively. Consequently, milk yield increased with increased amounts of SSL supplementation for the overall experimental period compared to that for the starting period (on-test yield; −2.19 in the CON group, −2.77 in the TRT 0.05 group, −1.18 in the TRT 0.1 group, and −0.8 kg in the TRT 0.2 group). *In vitro* ruminal studies [4–7,10] have demonstrated that supplemented non-ionic surfactants can enhance ruminal fermentation and improve feed efficiency due to their stimulatory effects on ruminal stability, ruminal microorganism numbers, growth performance, enzyme accessibility and synergy between cellulase and xylanase. For example, enhanced microbial growth can increase the amount of fibrolytic enzymes such as cellulase and xylanase, produced by rumen microorganisms, which could then increase acetate production as milk fat synthesis precursors increase and become converted to milk fat percentages. In our study, SSL supplementation (TRT 0.1) significantly improved the fat percentage compared to that of the CON and TRT 0.2 groups (4.15% vs 3.95% and 3.90%, respectively) and the fat yield compared to that of the CON, TRT 0.05, and TRT 0.2 groups (1.41 vs 1.29, 1.29 and 1.30 kg/d, respectively) (p<0.05). These results are consistent with those of previous studies by Dierick and Decuypere [25], demonstrating that SSL supplementation can significantly improve and promote milk fat synthesis to increase the fat percentage. Moreover, surfactant supplementation increased the amount of fatty acids from dietary lipids and improved their absorption in the small intestine. SSL supplementation (TRT 0.1) also improved the protein percentage compared to that of the TRT 0.2 group (3.48 vs 3.33 kg/d), which is consistent with the results of a previous study by Dickinson [26,27]. That study suggested that ionic surfactants had a relatively higher affinity for absorption by the interfacial protein, which could cause the protein network to be more easily disrupted, resulting in stronger electrostatic interactions than those formed by a non-ionic surfactant. Conversely, SSL supplementation (TRT 0.1) significantly reduced the MUN percentage compared to that of the CON, TRT 0.05 and TRT 0.2 groups (12.96% vs 14.27%, 14.89% and 14.49%, respectively) (p<0.05). This result suggests that the TRT 0.1 diet (35 g of SSL per 35 kg TMR) provided optimal microbial protein synthesis compared to the other treatments, even though all data were within the normal range (12 to 16 mg/dL) [28], indicating that sufficient carbohydrate amounts were provided for optimal rumen function and microbial synthesis [29]. However, SSL supplementation had no effect on the protein yield, lactose percentage, lactose yield or SNF content (p>0.10).

### Blood parameters

The hematological and biochemical blood parameters of mid-lactation cows supplemented with different SSL levels are shown in Table 3. Blood profiling is considered a significant indicator of nutrient status and body condition, and comparison of blood metabolites may help to diagnose and prevent metabolic disorders [30,31]. The most common blood diagnostic test is the complete blood count, which includes the WBC count, RBC count, hemoglobin content, and hematocrit value. No significant differences were observed among the treatments in the current study (p>0.05). All values were within the normal range, indicating that no significant changes were induced by the environmental and dietary adaptation of the cows [32]. The blood biochemical parameters, which evaluate the function of different organs including the kidneys and liver, and internal metabolic changes in cattle [33], showed no significant differences among the treatments (p>0.05). The exceptions were the albumin and ALT values, in which SSL supplementation via the TRT 0.05 diet (17.5 g of SSL per 35 kg TMR) resulted in lower albumin (3.9 g/dL) and higher ALT (48.5 IU/L) values than those of the CON group (4.1 g/dL and 39.8 IU/L, respectively) (p<0.05). However, the values of these measures were within the normal range as previously reported by Mohamed [34]. These results indicated that low-level SSL supplementation (TRT 0.05) might be insufficient to improve ruminal microbial protein synthesis and protein absorption, as shown by lower albumin values than those of the other treatments [35], and liver function of mid-lactation cows, as shown by the highest ALT value among the treatments [36]. The effect of SSL supplementation on hematological and biochemical parameters remains unclear.

### Economic efficacy

The economic efficacy of SSL supplementation is shown in Table 4. The total feed costs per head including TMR, concentrate and SSL were increased by 0.8%, 1.5%, and 3.1% for the TRT 0.05, TRT 0.1, and TRT 0.2 diets, respectively, compared to those in the CON diet. All milk samples were evaluated as grade 1A (in Korea), meaning that the raw milk included less than 30,000 bacterial cfu/mL and less than 200,000 somatic cells/mL (data not shown) [22]. Furthermore, milk yield and components (milk fat and protein content) are important for determining milk price and indicating whether animal health or nutritional management problems exist. Considering both the milk fat and protein content, the total milk price was set at 1,073.60 (TRT 0.05), 1,085.60 (TRT 0.1), 1,086.10 (TRT 0.2), and 1,064.20 (CON) won/L, with consequent total milk profits of −1.7%, 5.4%, and 3.5% for the TRT 0.05, TRT 0.1, and TRT 0.2 diet, respectively, compared to those in the CON diet. Based on these results, SSL supplementation (TRT 0.1) during the mid-lactation period of cows may increase profits.

## CONCLUSION

The results demonstrated that SSL supplementation using a TRT 0.1 diet could improve milk yield and milk composition (higher fat and protein content with a lower MUN concentration) compared to a CON diet. Consequently, the milk sales revenue related to SSL supplementation of the TRT 0.1 diet was increased by up to 5.4% compared to the milk sales revenue of the CON diet. Therefore, 0.1% SSL supplementation might be effective and profitable during the mid-lactation period of cows, without producing adverse effects. However, further study is needed to better understand how SSL supplementation will affect ruminal fermentation and blood metabolites in mid-lactation Holstein cows.

## Figures and Tables

**Table 1:** Ingredients and chemical composition of experimental total mixed ration (TMR) diets

Item	Value (g/kg)
Ingredients, as-fed basis	
Corn grain	20
Cotton seed	67
TMR granules	260
Soybean meal	37
Alfalfa hay	90
Beet pulp	60
Oat hay	100
Timothy hay	90
Limestone	2
Salt	2
NaHCO_3_	2
Water	270
Chemical composition, as-fed basis	
Dry matter	579.1
Crude protein	156.3
Crude fat	43.6
Crude fiber	192.3
Total ash	83.8
Calcium	8.9
Phosphorus	4.6
Neutral detergent fiber	414.6
Acid detergent fiber	218.7
Net energy of lactation (cal/kg)	1,560

**Table 2:** Effects of sodium stearoyl-2-lactylate (SSL) on milk yield and milk composition of Holstein cows

Item	Treatments1)				SEM

	CON	TRT 0.05	TRT 0.1	TRT 0.2	
Milk yield (kg/d)					
On-test yield	34.25	34.17	34.23	34.05	0.118
1 to 4 weeks	33.59	33.10	33.88	34.43	0.113
5 to 8 weeks	31.96^ab^	31.09^b^	32.98^ab^	33.28^a^	0.204
9 to 13 weeks	30.64^b^	30.01^b^	32.30^a^	32.59^a^	0.256
Overall	32.06^b^	31.40^b^	33.05^ab^	33.43^a^	0.189
Milk composition					
Fat (%)	3.95^b^	4.14^a^	4.15^a^	3.90^b^	0.026
Fat yield (kg/d)	1.29^b^	1.29^b^	1.40^a^	1.30^b^	0.011
Protein (%)	3.37^b^	3.50^a^	3.48^a^	3.33^b^	0.017
Protein yield (kg/d)	1.10	1.08	1.17	1.08	0.009
Lactose (%)	4.49	4.43	4.49	4.42	0.008
Lactose yield (kg/d)	1.46	1.36	1.51	1.39	0.014
SNF (%)	8.59	8.63	8.87	8.62	0.026
MUN (mg/dL)	14.27^a^	14.89^a^	12.96^b^	14.49^a^	0.171

SEM, standard error of the mean; SNF, solids-non-fat; MUN, milk urea nitrogen; TMR, total mixed ration.

1) Treatments: CON, basal diet; TRT 0.05, 17.5 g of SSL per 35 kg TMR; TRT 0.1, 35 g of SSL per 35 kg TMR; TRT 0.2, 70 g of SSL per 35 kg TMR.

ab Means with different superscripts within the same row differ (p<0.05).

**Table 3:** Effects sodium stearoyl-2-lactylate (SSL) on hematological and biochemical parameters of Holstein cows

Item	Treatments1)				SEM

	CON	TRT 0.05	TRT 0.1	TRT 0.2	
Hematological parameters					
White blood cells (K/μL)	12.6	13.9	10.6	15.2	0.40
Red blood cells (M/μL)	6.7	6.6	6.8	6.5	0.03
Hemoglobin (g/dL)	10.6	10.5	10.5	10.2	0.04
Hematocrit (g/dL)	30.8	30.5	30.3	29.2	0.14
Biochemical parameters					
Total protein (g/dL)	8.3	8.2	8.3	8.6	0.17
Albumin (g/dL)	4.1^a^	3.9^b^	4.0^ab^	4.1^a^	0.10
Glucose (mg/dL)	62.1	61.9	65.6	62.8	1.71
AST (IU/L)	91.6	103.8	93.1	94.4	5.50
ALT (IU/L)	39.8^b^	48.5^a^	40.1^b^	41.7^b^	4.07
GGT (mg/dL)	26.7	27.0	24.1	29.4	0.44
Total bilirubin (mg/dL)	0.3	0.3	0.4	0.3	0.09
BUN (mg/dL)	20.4	20.9	19.6	21.4	0.77
Creatinine (mg/dL)	0.9	0.9	0.9	0.9	0.01
Total glyceride, (mg/dL)	16.1	14.9	17.5	14.5	1.35
NEFA (μEq/L)	219.3	205.0	240.2	212.4	2.95
Cholesterol (mg/dL)	304.1	328.8	306.9	288.2	3.41

SEM, standard error of the mean; AST, aspartate aminotransferase; ALT, alanine aminotransferase; GGT, gamma glutamyl transferase; BUN, blood urea nitrogen; NEFA, non-esterified fatty acid; TMR, total mixed ration.

1) CON, basal diet; TRT 0.05, 17.5 g of SSL per 35 kg TMR; TRT 0.1, 35 g of SSL per 35 kg TMR; TRT 0.2, 70 g of SSL per 35 kg TMR.

ab Means with different superscripts within the same row differ (p<0.05).

**Table 4:** Effects of sodium stearoyl-2-lactylate (SSL) on the economic efficacy of Holstein cows

Item	Treatments1)			

	CON	TRT 0.05	TRT 0.1	TRT 0.2
A. Total feed cost (US $/305 d/head)	2,591.02	2,611.00	2,630.97	2,670.92
TMR	2,300.33	2,300.33	2,300.33	2,300.33
Concentrate	290.69	290.69	290.69	290.69
SSL	0.00	11.66	39.35	79.90
B. Total milk price (US $/305 d/head)	8,731.02	8,646.86	9,105.42	9,024.66
Milk yield	9,778	9,577	10,080	10,196
Milk price	0.89	0.90	0.90	0.89
B–A. Profit (US $/head)	6,140.00	6,035.86	6,474.45	6,353.74
Index (%)	100.0	98.3	105.4	103.5

TMR, total mixed ration.

1) CON, basal diet; TRT 0.05, 17.5 g of SSL per 35 kg TMR; TRT 0.1, 35 g of SSL per 35 kg TMR; TRT 0.2, 70 g of SSL per 35 kg TMR.
